# Surface ocean carbon dioxide variability in South Pacific boundary currents and Subantarctic waters

**DOI:** 10.1038/s41598-019-44109-2

**Published:** 2019-05-20

**Authors:** Paula C. Pardo, Bronte Tilbrook, Erik van Ooijen, Abraham Passmore, Craig Neill, Peter Jansen, Adrienne J. Sutton, Thomas W. Trull

**Affiliations:** 1 Antarctic Climate and Ecosystem Cooperative Research Center (ACE-CRC), Hobart, Australia; 2 CSIRO Oceans and Atmosphere, Hobart, Australia; 3 National Oceanic and Atmospheric Administration (NOAA)/Pacific Marine Environmental Laboratory (PMEL), Seattle, Washington USA

**Keywords:** Biogeochemistry, Carbon cycle

## Abstract

To improve estimates of the long-term response of the marine carbon system to climate change a better understanding of the seasonal and interannual variability is needed. We use high-frequency multi-year data at three locations identified as climate change hotspots: two sites located close to South Pacific boundary currents and one in the Subantarctic Zone (SAZ). We investigate and identify the main drivers involved in the seasonal an interannual (2012–2016) variability of the carbon system. The seasonal variability at boundary current sites is temporally different and highly controlled by sea surface temperature. Advection processes also play a significant role on the monthly changes of the carbon system at the western boundary current site. The interannual variability at these sites most likely responds to long-term variability in oceanic circulation ultimately related to climatic indices such as the El Niño Southern Oscillation, the Pacific Decadal Oscillation and the Southern Annular Mode (SAM). In the SAZ, advection and entrainment processes drive most of the seasonality, augmented by the action of biological processes in spring. Given the relevance of advection and entrainment processes at SAZ, the interannual variability is most probably modulated by changes in the regional winds linked to the variability of the SAM.

## Introduction

The ocean absorbs roughly 30% of the annual anthropogenic emissions of carbon dioxide (CO_2_)^1^. This uptake produces ocean acidification, which can have severe consequences for marine organisms and also reduces the ability of the ocean to uptake more CO_2_^2^. The amount of CO_2_ absorbed by the ocean varies across the globe and is modulated by several interrelated processes^3^. Sea-air exchange of CO_2_ depends primarily on the difference of partial pressures of CO_2_ between the air and the sea surface and on the wind speed. Biological processes also condition this exchange via photosynthesis and respiration. Oceanic circulation drives the redistribution of the surface ocean carbon and its vertical exchange with the deep ocean, thus also affecting the ocean-atmosphere carbon fluxes through changes in surface ocean content. Altogether, these processes imprint spatial, seasonal and interannual variability on the marine carbon system^3^.

This natural variability of the marine carbon system can be very high, making the detection of long-term trends difficult^4^. An improved understanding of the drivers of sub-seasonal to interannual variability is an important part of predicting the future ocean and how the carbon cycle will respond to a changing environment. The increase in the number and diversity of platforms delivering high-frequency data such as gliders, floats, ocean moorings or satellites^5–7^ is helping to deliver new data to understand the drivers of the upper ocean CO_2_ variability. Time series moorings, with an expanding range of sensors, are a powerful tool for estimating atmospheric and oceanic carbon changes on seasonal to long-term timescales^8^. The most recent studies on the variability of the marine carbon uptake^9,10^ have been possible thanks to the development of internationally coordinated observing networks in which moorings are a fundamental component. These studies point out the relevance of the seasonal and short-term variability of the carbon system to the detection of long-term trends and the complexity of identifying the main drivers of the seasonal and spatial variability.

**Figure 1:**
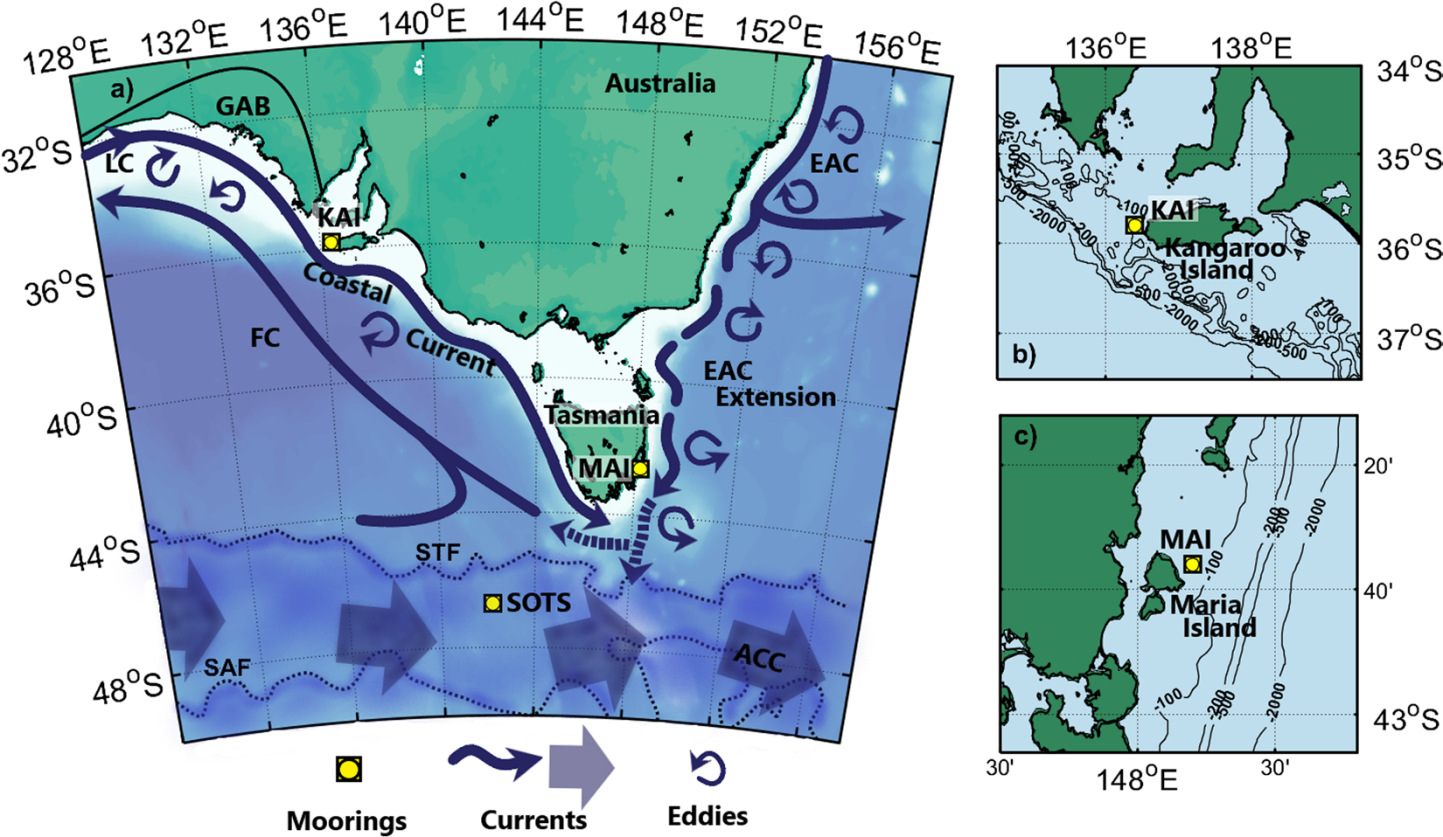
Oceanographic settings. (**a**) Locations of time series mooring sites and associated geographic and hydrodynamic features. Time series: Kangaroo Island (KAI, zoomed in plot **b**), Maria Island (MAI, zoomed in plot **c**) and Southern Ocean Time Series (SOTS). Features: Leewin Current (LC), Great Australian Bight (GAB), Coastal Current, Flinders Current (FC), Antarctic Circumpolar Current (ACC), Subtropical Front (STF; estimated from MODIS Aqua sea surface temperature^67^) and Subantarctic Front (SAF; estimated from sea surface height^68^), East Australian Current (EAC) and EAC extension.

We describe the variability and drivers of surface-ocean fugacity ($fC{O}_{2}^{sw}$) by using high-frequency data from three moorings located south of Australia: Kangaroo Island (KAI), Maria Island (MAI) and the Southern Ocean (SOTS) (Fig. 1). These moorings are capable of climate quality measurements and are strategically located in climate change hotspots South of Australia: in the Subantarctic Zone (SAZ) and at South Pacific boundary currents. Boundary current systems play crucial roles in transport of heat, water masses and other properties^11^, strongly affecting regional climate and ecosystems and the SAZ is the most important region of the Southern Ocean in terms of CO_2_ uptake^12^. The data are integrated into global networks^6,13^ and are used for validation and assimilation in current analytical and predictive models^14^. We also analyse ancillary data from hydrographic lines, satellites, and model runs to give more insight into the biological processes and ocean dynamics affecting $fC{O}_{2}^{sw}$ variability at seasonal scales. Furthermore, we investigate the interannual variability of $fC{O}_{2}^{sw}$, elucidating its main drivers and possible future implications for long-term trends.

## Oceanographic Settings

KAI (36°S, 136°E) is located on the southern Australian shelf in 105 m of water to the east of the Great Australian Bight (GAB, Fig. 1). The shelf is the largest actively growing carbonate platform on earth and is a region with ecosystems of high ecological and economic importance^15^. The oceanography of the region is highly dynamic, with a seasonal variability linked to the passage of high pressure systems^16^. South-easterly winds in the austral summer (Dec-Mar) transport waters from the Southern Ocean and eastern Tasmania and along the continental slope of the GAB as the Flinders Current^16^ (FC, Fig. 1). The winds are favourable to upwelling during these months and drive episodic upwelling events of relatively cold offshore waters onto localized areas of the continental shelf^17^ (mostly south of Kangaroo Island), which are essential to the maintenance of the shelf ecosystems^15^. A shift in the winds towards autumn (Mar-Jun) drives the development of an eastward flowing coastal current along the continental shelf (Fig. 1) that suppresses upwelling, and is strongest in winter (Jun-Sep)^18^. This coastal current is a mixture of waters from the Leeuwin Current (LC, Fig. 1) and entrained shelf waters^16,19^. The LC is the poleward eastern boundary current that flows down the west coast of Australia before turning east and flowing along the southern Australian shelf. The projection of the LC along the southern shelf is strongest during winter when it contributes to the extension and persistence of the coastal current^16^. The link of the LC and the coastal current to the atmospheric systems makes them especially sensitive to climate change, which could change the dynamics of the area, alter the water properties and have diverse impacts on the fisheries and marine organisms^20^.

MAI (43°S, 148°E) is located off the east coast of Tasmania in 85 m of water of the South Tasman Sea and is also located in the extensive carbonate producing platform of the southern Australia shelf^15^. The circulation of this region is dominated by the boundary current system of Eastern Australia: the East Australian Current (EAC, Fig. 1). The EAC originates off northeastern Australia at ~20°S, and is fed by waters from the South Pacific subtropical gyre^21^. Near 33–34°S, much of its transport separates from the coast and turns east but a highly-variable tongue, the EAC extension (Fig. 1), of relatively warm and salty EAC waters extends southward along the East Australian coast as a series of energetic mesoscale features^21^. The EAC extension creates a zonal jet south of Tasmania that connects Pacific and Indian waters, as part of the global thermohaline circulation. Most of this flow follows a northwest direction off southern Tasmania, as part of the FC and the rest goes southward in the form of eddies^22^. The EAC extension is strongest in the austral summer and weakens over winter allowing cooler and fresher modified subantarctic surface waters to spread north and into the vicinity of MAI^23^. The seasonality of the EAC extension influences the regional climate as well as the marine living systems and fisheries of the area near MAI^24^. The region east of Tasmania has shown the highest warming rates observed in the last seven decades in the Southern Hemisphere and the strength of the EAC extension is predicted to increase under future climate scenarios with important implications for fisheries, the marine carbon system and other oceanic processes^20^.

SOTS (47°S, 142°E) is in >4500 m of water in the SAZ, the region of the Southern Ocean between the Subtropical (STF) and Subantarctic (SAF) fronts of the Antarctic Circumpolar Current (ACC)^25^ (Fig. 1). The Southern Ocean is the largest net oceanic sink of atmospheric CO_2_ and the SAZ is the area responsible for most of this uptake^12^. The waters of the SAZ are influenced by many dynamic processes such as the ACC flow, the mixing between subtropical and subantarctic waters, the subduction of subsurface waters as part of the upper cell of the oceanic thermohaline circulation and the spawning of eddies from the SAF associated with meanders in the front due to the interaction of the SAF with the bathymetry^25^. The SAZ to the south of Tasmania is also affected by the EAC extension^22^ (Fig. 1). The interaction between subantarctic and subtropical waters are believed to be contributors to high seasonal primary production in the SAZ^26^. Over recent decades, numerous studies have shown long time-scale variability of the Southern Ocean in terms of atmospheric carbon uptake^10,27^ although data from this area is scarce with SOTS being one of the only two high-frequency surface time-series sites located in the Southern Ocean^28^.

**Figure 2:**
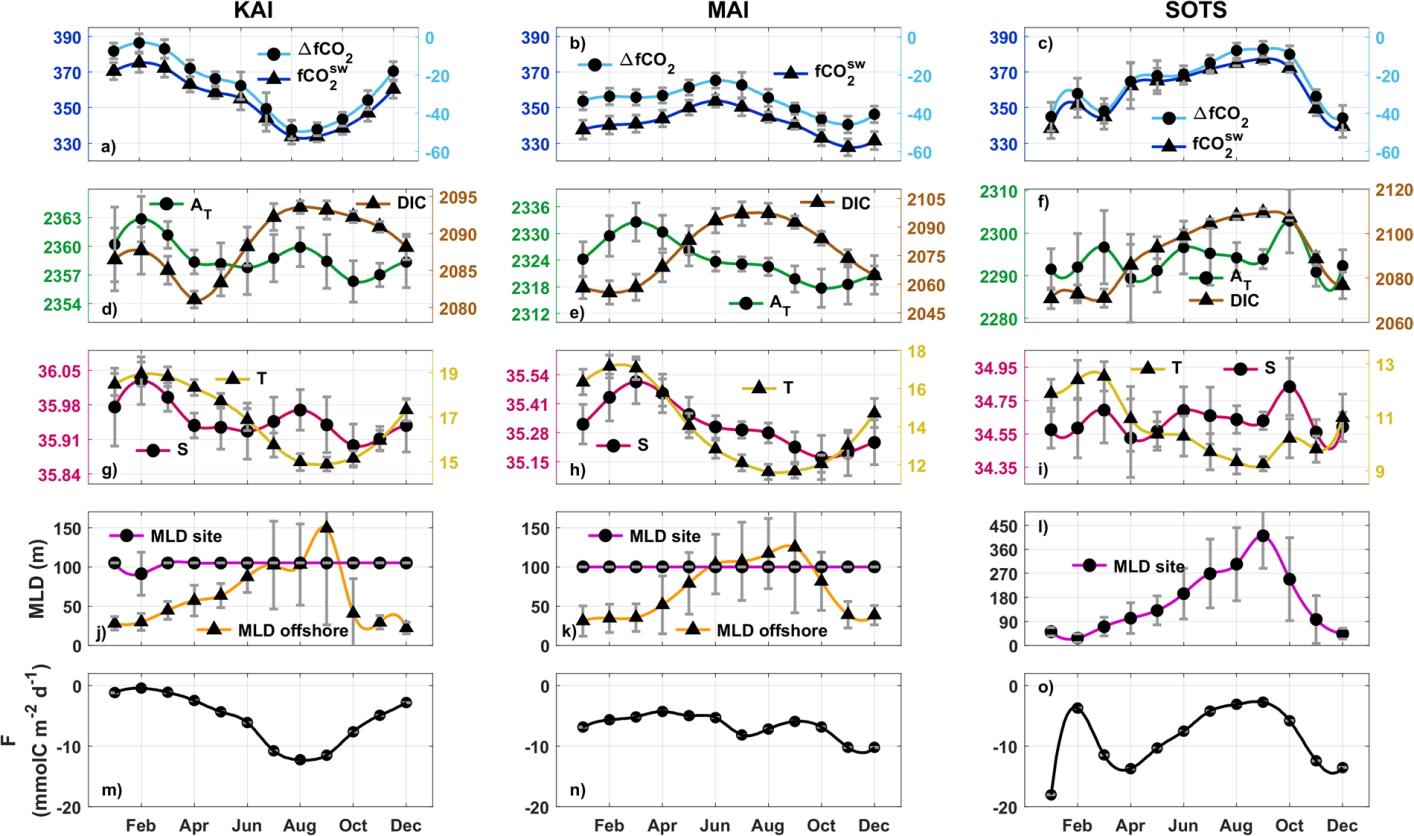
Seasonal variability at each site of: (**a**–**c**) sea-surface CO_2_ fugacity ($fC{O}_{2}^{sw}$, dark-blue line with triangles) and difference respect to the atmosphere (${\rm{\Delta }}fC{O}_{2}$, light-blue line with circles); (**d**–**f** ) total alkalinity (A_T_, green line with circles) and dissolved inorganic carbon (DIC, brown line with triangles), both in μmol kg^−1^; (**g**–**i**) salinity (S, psu, magenta line with circles) and temperature (T, Celsius, yellow line with triangles); (**j**–**l**) mixed layer depth (MLD, meters) at the mooring locations (fuchsia line with circles) and off-shore (orange line with triangles); (**m**–**o**) air-sea CO_2_ fluxes (F, black line with circles, negative values indicate fluxes into the sea).

The oceanographic settings at the three sites influence the mean de-trended (Eq. 1) annual values and the seasonal amplitude of temperature and salinity (Fig. 2g–i). The boundary current sites (KAI and MAI) are characterized by warmer and saltier waters with a greater subtropical component than the higher latitude site (SOTS) that is mainly influenced by subantarctic waters. At KAI the influence of South Indian subtropical waters combined with local inputs from the shelf^19^ results in higher salinity waters than at the MAI site, which is influenced by western South Pacific subtropical waters^29^ (Fig. 2g,h). The seasonal changes in salinity at KAI (Fig. 2g) are less than at MAI and SOTS as these latter two sites have greater amounts of warm and salty subtropical waters present in warmer months and more subantarctic waters in the cooler months (Fig. 2g–i). These differences also extend to the mean annual values of total alkalinity (A_T_), which is related to salinity, and dissolved inorganic carbon (DIC) that decrease and increase, respectively, at higher latitudes (Fig. 2d–f).

## Seasonal Variability of ${\boldsymbol{fC}}{{\boldsymbol{O}}}_{2}^{{\boldsymbol{sw}}}$and Mean Drivers

The de-trended seasonal cycles (second and third terms of Eq. 1) for $fC{O}_{2}^{sw}$ are temporally different for the three sites, while the annual mean values and the seasonal amplitudes are similar (Fig. 2a–c). All three sites have negative sea-air gradients in $fC{O}_{2}$ (${\rm{\Delta }}fC{O}_{2}=fC{O}_{2}^{sw}-fC{O}_{2}^{atm}$) (Fig. 2a–c) for most of the year and are sites of net annual uptake of CO_2_ from the atmosphere (Fig. 2m–o).

**Figure 3:**
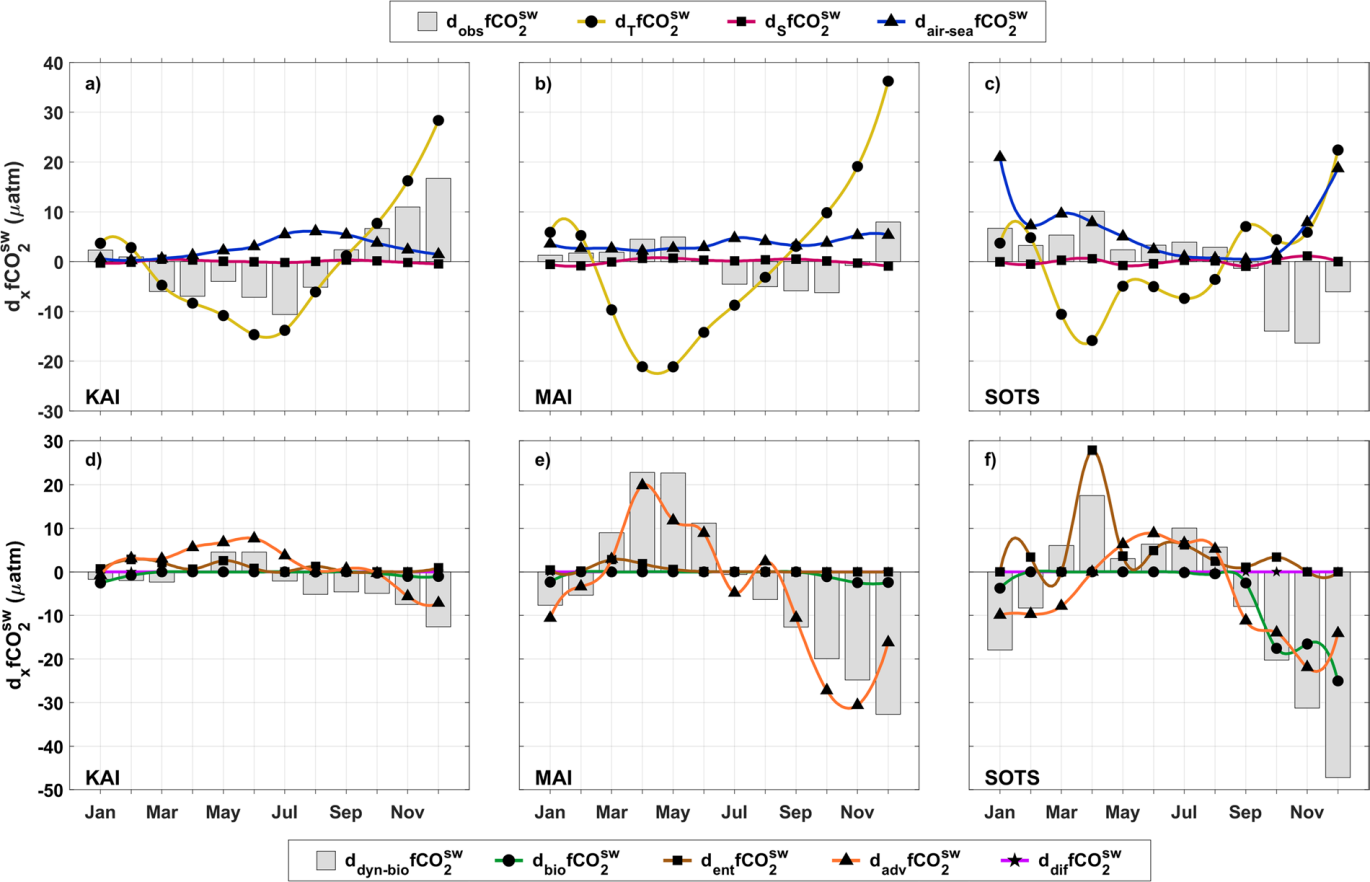
Attribution of monthly CO_2_ fugacity changes across physical and biological drivers at each site. (**a**–**c**) measured monthly changes in $fC{O}_{2}^{sw}$ (${d}_{obs}\,fC{O}_{2}^{sw}$, grey boxes) and expected changes in $fC{O}_{2}^{sw}$ due to the variability of temperature (${d}_{T}fC{O}_{2}^{sw}$, yellow line with circles), salinity (${d}_{S}\,fC{O}_{2}^{sw}$, magenta line with squares) and air-sea fluxes (${d}_{air-sea}\,fC{O}_{2}^{sw}$, blue line with triangles); (**e**–**g**) estimated changes in $fC{O}_{2}^{sw}$ due to the combined effect of dynamics and biological processes (${d}_{dyn-bio}\,fC{O}_{2}^{sw}$, grey boxes) and separately due to primary productivity (${d}_{bio}\,fC{O}_{2}^{sw}$, green line with dots), advection (${d}_{adv}\,fC{O}_{2}^{sw}$, orange line with triangles), entrainment (${d}_{entr}\,fC{O}_{2}^{sw}$, brown line with squares) and diffusion (${d}_{dif}\,fC{O}_{2}^{sw}$, fuchsia line with stars)(see Eqs 2 and 3 in Methods).

At KAI, $fC{O}_{2}^{sw}$ approaches near-equilibrium values with the atmosphere in late summer (Feb, ${\rm{\Delta }}fC{O}_{2}$ = −3.1 ± 5.2 μatm), decreases through winter to a minimum in about Aug (${\rm{\Delta }}fC{O}_{2}$ = −49 ± 4.4 μatm), and increases over the spring-summer to near equilibrium values (Fig. 2a). The rate of change of $fC{O}_{2}^{sw}$ (${d}_{obs}\,fC{O}_{2}^{sw}$, Fig. 3a) is between −5 and −10 μatm per month from early autumn (Mar) through winter (Aug), before becoming positive from spring until early summer (Dec) (Fig. 3a). At MAI, the $fC{O}_{2}^{sw}$ seasonality is about four months out of phase with that of KAI, with a minimum in Nov and a maximum in Feb and about half the seasonal amplitude as the other sites (Fig. 2b). The smallest sea-air gradient at MAI occurs during winter (${\rm{\Delta }}fC{O}_{2}$ = −23.4 ± 3.5 μatm) and does not approach equilibrium with the atmosphere as observed at the other sites (Fig. 2c). The monthly rate of change at MAI is also relatively small and typically less than ±5 μatm for most of the year (Fig. 3b). The seasonal amplitude of $fC{O}_{2}^{sw}$ at SOTS (Fig. 2c) is similar to KAI, with the maximum value close to equilibrium with the atmosphere (${\rm{\Delta }}fC{O}_{2}$ = −8.9 ± 6 μatm). However, $fC{O}_{2}^{sw}$ at SOTS is about 6 months out of phase with KAI and is characterized by a rapid decrease from a maximum in Sep to a minimum in Dec with a more gradual increase from summer through the end of winter (Fig. 3c).

The differences in the de-trended seasonal changes of $fC{O}_{2}^{sw}$ at the sites are due to shifts in the timing and relative importance of the physical and biological drivers (Eqs 2 and 3). To describe these differences, first we computed (Eq. 2) the changes in $fC{O}_{2}^{sw}$ due to sea-air uptake (${d}_{air-sea}\,fC{O}_{2}^{sw}$) and changes in sea surface temperature (${d}_{T}fC{O}_{2}^{sw}$) and salinity (${d}_{T}fC{O}_{2}^{sw}$) and compared them with the values of ${d}_{obs}\,fC{O}_{2}^{sw}$ (Fig. 3a–c). Then, we compared the residual of Eq. 2, i.e. ${d}_{{\rm{dyn}}-{\rm{bio}}}\,fC{O}_{2}^{sw}$, with the changes in $fC{O}_{2}^{sw}$ due to primary production (${d}_{bio}\,fC{O}_{2}^{sw}$), entrainment (${d}_{entr}fC{O}_{2}^{sw}$), advection (${d}_{adv}\,fC{O}_{2}^{sw}$) and diffusion (${d}_{dif}\,fC{O}_{2}^{sw}$) (see Eq. 3 and Fig. 3d–f). We describe the role of the main drivers for the different seasons considering the uncertainties of the different terms (see Methods). The effect of changes in salinity and diffusion have been left out of the discussion since their effects on ${d}_{obs}\,fC{O}_{2}^{sw}$are negligible (Fig. 3).

**Figure 4:**
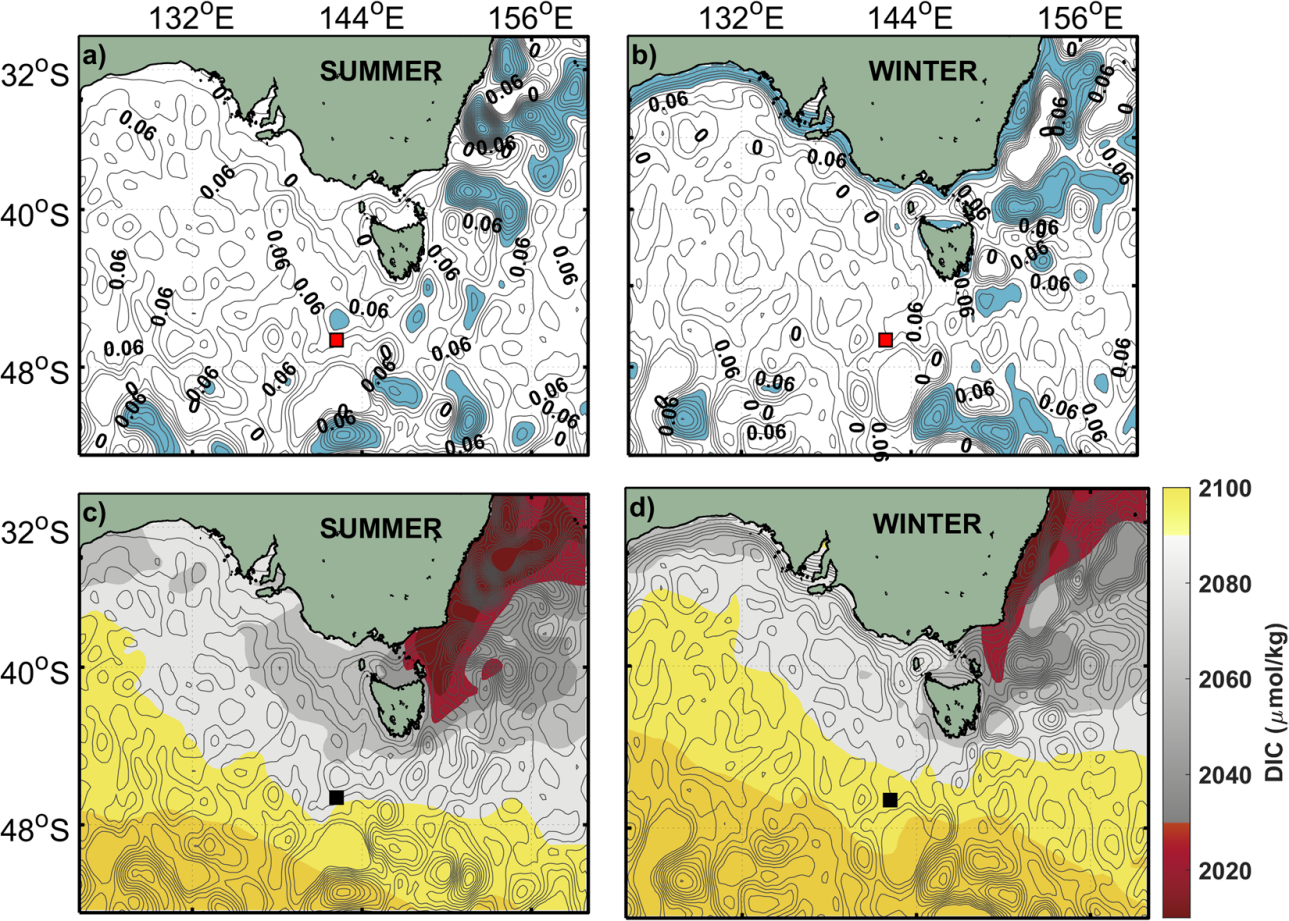
Seasonal changes in circulation south of Australia. (**a,b**) mean sea level anomalies (SLA) in meters in (**a**) summer and (**b**) winter, SLA >0.06 m in blue highlights the strong winter-time coastal currents along the Australian shelf, and the summer-time intensification of the EAC extension; (**c**,**d**) distribution of surface-ocean DIC in (**c**) summer and (**d**) winter. The SOTS location is indicated by red squares in (**a**), (**b**) and black squares in (**c**), (**d**) SLA distribution from IMOS OceanCurrent database^46^ and DIC distribution from B-SOSE products^14^.

At KAI, the seasonal cycles of temperature and $fC{O}_{2}^{sw}$ show similar patterns (Fig. 2a,g). From autumn to winter (Mar-Sep), the cooling of surface waters decreases $fC{O}_{2}^{sw}$ (${d}_{T}fC{O}_{2}^{sw}$, Fig. 3a). In autumn (Mar-Jun), regional winds pile water up along the coast favouring the development of the coastal current^16^ (Fig. 4). This retraction of shelf waters to the coast promotes the northward transport or advection of water coming from offshore^19,30^. The offshore waters are modified subantarctic waters including FC water and cause a decrease in temperature, salinity and A_T_, while DIC increases (Fig. 4d). The increase of DIC by advection (Fig. 3d) partially counteracts the effect of temperature (Fig. 3a). A progressive deepening of the mixed layer (MLD, Fig. 2j) due to the strengthening of the winds allows DIC-rich deeper water to be entrained into the mixed layer tending to increase $fC{O}_{2}^{sw}$, although the net effect of the entrainment is small (at the limit of its uncertainty, Fig. 3d).

In winter, a combination of stronger winds and more negative ${\rm{\Delta }}fC{O}_{2}$ increases the net flux into the ocean (Fig. 2m) and $fC{O}_{2}^{sw}\,$(${d}_{air-sea}\,fC{O}_{2}^{sw}$, Fig. 3a). The opposing effect of decreasing temperature prevails and the values of $fC{O}_{2}^{sw}$ continue to decrease through the winter. In July-August, the coastal current peaks (Fig. 4b) and widens^16,30^, affecting the area around KAI and causes an increase of salinity and A_T_ (Fig. 2d,g). The coastal current dissipates quickly towards the end of winter^19^ and over Dec-Feb the advection of warmer and saltier subtropical Indian waters, with relatively high A_T_ and low DIC could possibly act to limit the increase of $fC{O}_{2}^{sw}$ caused by warming surface waters (Fig. 3a,d, notice that the changes in ${d}_{{\rm{dyn}}-{\rm{bio}}}\,fC{O}_{2}^{sw}$ are almost below its uncertainty for the first part of the year). For the KAI region, the relative increase in salinity and A_T_ during summer may also be related to the outflow from the nearby coastal embayments^31^ due to the relaxation of the coastal current and the prevailing upwelling-favourable winds. Upwelling does occur in the vicinity of KAI, although the upwelling at the site remains subsurface^32^ and the effect on surface-layer primary production and $fC{O}_{2}^{sw}$ is negligible (Fig. 3d).

At MAI, the role of temperature on $fC{O}_{2}^{sw}$ (${d}_{T}fC{O}_{2}^{sw}$, Fig. 3b) is counteracted by the seasonal extension and contraction of subtropical waters associated with the EAC extension. During autumn and winter, a greater proportion of modified subantarctic waters occur at the site^23^ causing an increase in DIC (Fig. 4) and decreases in salinity, A_T_, and temperature (Fig. 2e,h). The action of advection increases $fC{O}_{2}^{sw}$, offsetting the effect of decreasing temperature in autumn (Fig. 3b,e). The impact of advection of modified subantarctic waters diminishes over winter and the seasonal cooling leads to a decrease in $fC{O}_{2}^{sw}$ (Fig. 3b,e). From spring to summer, the EAC extension intensifies^23^ (Fig. 4a), bringing subtropical waters to the area with relatively higher A_T_ and lower DIC (Fig. 2h,e). The increased transport of the subtropical waters into the region during spring offsets the warming effect and causes a slight decrease in $fC{O}_{2}^{sw}$. Our calculations indicate that the effect of the biological driven changes (${d}_{bio}\,fC{O}_{2}^{sw}$) at MAI is negligible during this period, which is consistent with the reported low to moderate primary production in the region^32^.

At SOTS, the net sea-air fluxes (${d}_{air-sea}\,fC{O}_{2}^{sw}$, Fig. 3c) have a more significant effect on the variability of $fC{O}_{2}^{sw}$ than at the two shelf sites and can approach the influence of temperature (${d}_{T}fC{O}_{2}^{sw}$). The SAZ is one of the most important areas in terms of oceanic carbon uptake^12^ and it can be seen in the values of the sea-air fluxes that achieve the highest negative values of all the locations (Fig. 2o). The high sea-air fluxes are favoured by the cold subantarctic waters and strong winds that are characteristic of this region^25^. The strengthening of winds also deepens the mixed layer at SOTS (Fig. 2l) mixing high DIC waters into the surface layer and increases the entrainment term in autumn (${d}_{entr}fC{O}_{2}^{sw}$, Fig. 3f). The sea-air fluxes and entrainment cause most of the increase in $fC{O}_{2}^{sw}$, and exceed the decrease in $fC{O}_{2}^{sw}\,$due to cooling (Fig. 3c,f). The impact of the sea-air fluxes, temperature and entrainment diminishes through winter, while the role of the advection, that is related to the northward transport of DIC-rich subantarctic waters^33^ increases and acts to maintain the increase of $fC{O}_{2}^{sw}$ (Fig. 3c,f).

In spring at SOTS, the seasonal increase of temperature tends to increase $fC{O}_{2}^{sw}\,$while advection and primary productivity cause a decrease in $fC{O}_{2}^{sw}$ (Fig. 2c,f). The advection processes during this season are related to the arrival, in the form of eddies^22^, of subtropical waters (with low DIC and high salinity and A_T_) that mix with subantarctic waters. The impact of primary production during spring is comparable to that of advection (Fig. 3f). Previous studies at SOTS and the Southern Ocean have highlighted the relevance of the primary production and its effect on the carbon cycle during spring to early-summer^10,34^. Over summer, the effect of the primary production and advection diminishes and the combined action of the sea-air fluxes, temperature and entrainment results in a net increase of $fC{O}_{2}^{sw}$ (Fig. 3c,f).

## Interannual Variability of ${\boldsymbol{fC}}{{\boldsymbol{O}}}_{2}^{{\boldsymbol{sw}}}$

**Figure 5:**
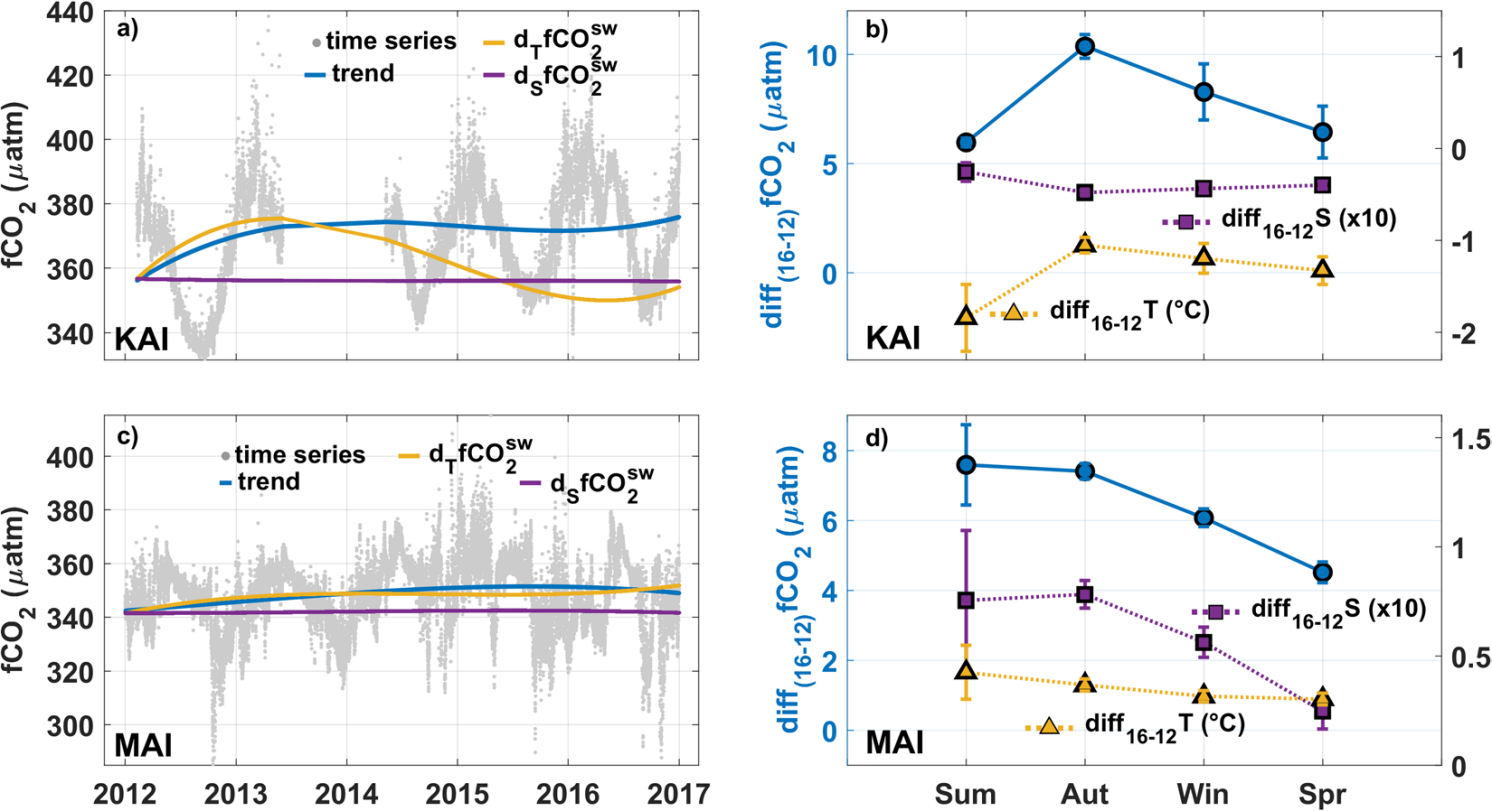
Interannual variability in the carbon system at MAI and KAI. (**a**,**b**) Original time records between 2012 and 2016 (grey dots) and interannual trend (blue line) of $fC{O}_{2}^{sw}$ together with the contributions to the interannual trend of $fC{O}_{2}^{sw}\,$by the interannual changes in temperature(${d}_{T}fC{O}_{2}^{sw}$, yellow line) and salinity (${d}_{S}\,fC{O}_{2}^{sw}$, magenta line); (**b**,**d**) cumulative changes in $fC{O}_{2}^{sw}$ between 2012 and 2016 (blue line with dots), temperature (yellow line with triangles) and salinity (magenta line with squares) shown as averages in each season (Sum = summer, Aut = autumn, Win = winter, Spr = spring). The seasons correspond to the Southern Hemisphere and are defined using celestial boundaries. The values of the cumulative changes in salinity and temperature are represented in the right Y-axis. The values of the changes in salinity have been multiplied by ten.

There is significant interannual variability superimposed on the seasonal changes described above indicating that there are additional processes acting at longer than seasonal time scales that are influencing the CO_2_ variability^9^. In order to assess the interannual variability we used de-seasonalized data (first term in Eq. 1) from KAI and MAI. We contrasted the interannual variability at KAI and MAI between 2012 and 2016. This period was covered by the data at both sites, with the exception of the period from Jun-2013 to May-2014 at KAI when no data were collected (Fig. 5). The SOTS data contained a number of gaps that doesn’t allow for a similar assessment at this site (see Methods).

At KAI, the de-seasonalized series (trend in Fig. 5a) can be approximated to a linear trend of ~2.0 ± 0.6 μatm yr^−1^ for the period 2012–2016, which would indicate that the increase in $fC{O}_{2}^{sw}$ at this site is lower than expected if surface waters tracked the atmospheric CO_2_ increase^35^ of ~2.5 ± 0.2 μatm yr^−1^. This is not surprising since recent studies have pointed out the difficulty of separating the anthropogenic signal from the natural variability in the air-sea carbon fluxes^4^ with the time of emergence of the anthropogenic signal in certain areas of the ocean being higher than three decades. The 5-year trend in salinity is small (−0.0103 ± 0.0001 y^−1^) and has little effect on $fC{O}_{2}^{sw}$ over the period of measurements $({d}_{S}\,fC{O}_{2}^{sw}$, Fig. 5a). The observed interannual change in sea-surface temperature (${d}_{T}fC{O}_{2}^{sw}$) over the period corresponds to a net 5-year decrease of −0.3183 ± 0.0017 °C y^−1^ and has a stronger effect than salinity. The cumulative monthly change in the de-seasonalized values of $fC{O}_{2}^{sw}$ between 2012 and 2016 (Fig. 5b) indicates that most of the change occurs in autumn and winter. The corresponding monthly changes of salinity and temperature show similar patterns to that of $fC{O}_{2}^{sw}$, with salinity showing a higher decrease and temperature a lower decrease in autumn (Fig. 5b). The similarity in the monthly changes in salinity and temperature suggests that the 5-year change in $fC{O}_{2}^{sw}$ is not only driven by the atmospheric CO_2_ increase and the temperature changes but also by changes in the regional circulation.

Results from observation analysis and high resolution modelling suggest that an increase in the transport and eddy kinetic energy of the LC and FC driven by a wind change has occurred in response to a climate regime shift in the Pacific^20,36,37^. This change in the Pacific climate regime has been linked to the variability of major sources of inter- and intra-decadal global climate variability, in particular, the El Niño Southern Oscillation (ENSO) and Pacific Decadal Oscillation (PDO)^20,36.^ Cold periods of PDO, such as it occurred during the observational period^20^, are associated with the poleward extension of the EAC and the enhancement of wind curls over South Australia^36^. The poleward extension of the EAC would imply an increase in the contribution of East Tasmanian waters to the FC which, added to the wind curl increase, would result in the enhancement of the volume transport of the FC into the region^20^. An enhanced FC could bring DIC-rich cooler waters from the south to the KAI region that would produce a general cooling, freshening and an increase in DIC and $fC{O}_{2}^{sw}$ especially in summer, when the coastal current is weaker (Fig. 5b). During the observational period, the ocean was under La Niña conditions that are linked to an increase in the volume transport of the LC^36,37^ and consequently its intrusions into the GAB in autumn and winter (Fig. 1). In addition, the invigoration of the LC has been associated with anomalous warming and freshening^38,39^. The warming and freshening of the LC flow would explain the higher increase of $fC{O}_{2}^{sw}$ as well as the relative lower decrease of temperature observed during autumn and winter and the relative higher decrease of salinity in autumn (Fig. 5b).

At MAI, the interannual increase in $fC{O}_{2}^{sw}$ between 2012 and 2016 (trend in Fig. 5c) can be linearly approximated to a value of 1.7 ± 0.3 μatm yr^−1^, which is less than at KAI, and is also less than the increase expected due to the increase in atmospheric CO_2_. The 5-year trend in salinity corresponds to an increase of 0.0174 ± 0.0001 y^−1^ and does not have a significant effect on $fC{O}_{2}^{sw}$ (Fig. 5c). The temperature increase for the period 2012–2016 (0.0745 ± 0.0003 °C y^−1^) dominates the interannual variability of $fC{O}_{2}^{sw}$ limiting a direct response to the atmospheric CO_2_ increase (Fig. 5c). The net monthly increase in $fC{O}_{2}^{sw}$ at KAI between 2012 and 2016 is stronger in summer and autumn in agreement with the highest increases in temperature and salinity for this period (Fig. 5d).

A long-term warming has occurred at MAI over the last seven decades due to a poleward shift and intensification in the transport and eddy kinetic energy of the EAC^20,40,41^. The EAC strengthening and poleward shifting has been related to the multi-decadal spin-up of the Pacific subtropical gyre, associated with the variability of the PDO and ENSO^20,40,41^. This intensification is transferred into the EAC extension as an increased transport of warm-core eddies that also bring low-DIC waters into the region displacing relatively cooler and high-DIC subantarctic waters. The EAC extension is greatest in summer to early autumn and coincides with the largest increase in $fC{O}_{2}^{sw}$ found for the period 2012–2016 (Fig. 5d) that is linked to the warming influence of the EAC on $fC{O}_{2}^{sw}$. Although small, the general increase in salinity (Fig. 5d) also supports our reasoning of an increase in the component of subtropical waters at the MAI region due to the intensification of the EAC extension.

## Summary and Global Connections

Changes in volume transport, poleward extension and warming rates at oceanic boundary currents have been linked to shifts in climate regimes over large areas of the ocean^20,36^. These climate regime shifts are ultimately related to inter- and intra-decadal variability of climate indices such as the PDO, ENSO or the Southern Annular Mode (SAM)^36^.

**Figure 6:**
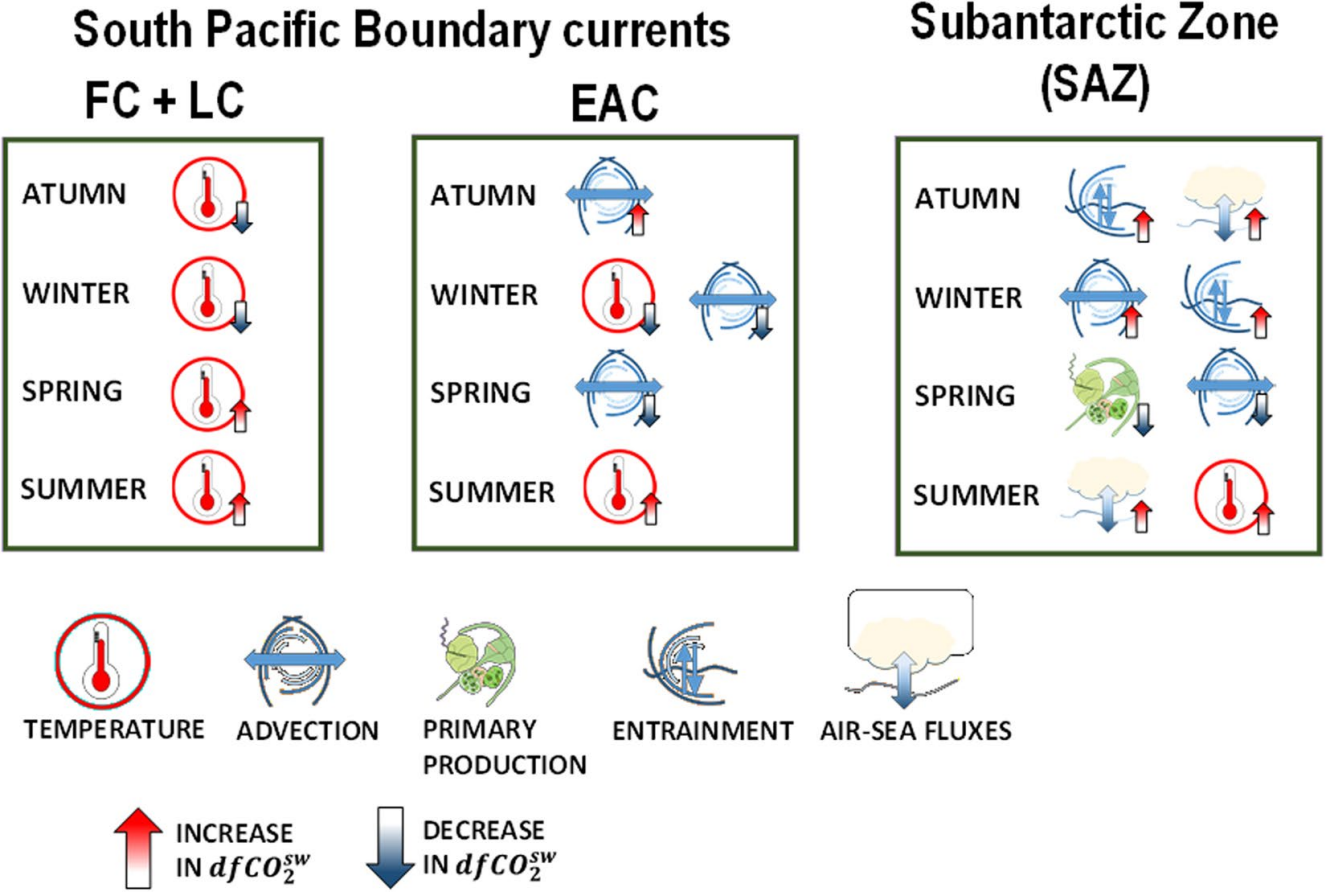
Summary of the main drivers responsible for the seasonality of $fC{O}_{2}^{sw}$ in regions of the South Pacific boundary currents and the SAZ. FC = Flinders Current, LC = Leewin Current and EAC = East Australian Current.

Our results for the South Pacific boundary current sites (KAI, MAI) show that the seasonal variability of the carbon cycle is temporally different between regions but mainly controlled by changes in sea surface temperature (Fig. 6). Advection processes have also a relevant role on the seasonal variability of $fC{O}_{2}^{sw}$, specifically for west boundary currents, such as the EAC (Fig. 6). The warming associated with boundary currents, that is expected to increase in future scenarios^36^, would consequently have a relevant effect on the carbon system of these and other similar regions. The most probable change will be a reinforcement of the increase in the amplitude of the seasonal variability that has been observed for more than 30 years^42^. Some hints of this were noticed in the mathematical fit done to separate seasonal from multi-year variability (Eq. 1), where adding the variability in the seasonal amplification improved the fit. Unfortunately, the time coverage of the time series is too short to allow for a deeper discussion on this issue.

The variability of climatic indices has a major role on the interannual variability of $fC{O}_{2}^{sw}$, lowering the expected response of the carbon system to the anthropogenic increase of atmospheric CO_2_. This strong role of the multiyear atmosphere-ocean variability indicates that the variability over a 5-year period is only representative of this period and that the interannual variability in a future 5-year period could be totally different if the climatic indices were under new phases. This result is a good motivation for maintaining long-term high-frequency oceanic observations to elucidate better the response of the marine carbon system to the atmospheric CO_2_ increase.

In the SAZ, the seasonal variability of $fC{O}_{2}^{sw}$ is mainly controlled by advection and entrainment processes, with an important role of biological processes in spring (Fig. 6). The processes of entrainment and advection in the SAZ are modulated by the action of the westerly winds over the region, closely linked to the variability of the SAM^33,43,44^. Observation analysis have already shown the link between the SAM and changes in the surface ocean carbon system of the Southern Ocean^10,45^. Changes in surface ocean properties have also being considered the most probable cause behind the increase in the amplitude of the seasonal variability^42^. Climate models predict intensification of the westerlies and enhanced warming under a more positive phase of the SAM^36^, suggesting that the amplitude of the seasonal variability of $fC{O}_{2}^{sw}$ is likely to increase. Nevertheless, this conclusion remains difficult to foresee due to the high uncertainties in the climatic projections^36^ and in the data-limited observational analysis and the different relative weights of the drivers in the seasonal variability (Fig. 6).

## Methods

High frequency (~3-hourly) surface measurements of salinity (S), temperature (T) and the mole fraction of CO_2_ (XCO_2_) in atmospheric air and air equilibrated with surface seawater were obtained from autonomous sensors at each mooring site using SBE16PlusV2 (from Seabird Electronics Inc) and MapCO2 sensors^6^. The moorings and sensors were replaced approximately every six months. KAI data are from Feb-2012 to May-2017 with a gap in observations from Jun-2013 to May-2014. MAI data cover the period from Apr-2011 to Sep-2017. The remote location and conditions at the SOTS site have resulted in a number of gaps with data available from Nov-2011 to Sep-2012, the last six months of 2013 and three months (autumn to winter) in 2016.

$fC{O}_{2}^{sw}$ was calculated from XCO_2_ at T, S and pressure at the sea surface. The accuracy of $fC{O}_{2}^{sw}$ was estimated to be ±2 μatm in all moorings^6^ and verified against zero CO_2_ and reference CO_2_ gas samples run before each 3 hr sampling point. Laboratory validations of the MapCO2 systems before deployment and after retrieval of the sensors confirmed measurement accuracy. S and T sensors calibrations were also confirmed before deployment and after retrieval at a National Association of Testing Authorities facility at CSIRO Oceans and Atmosphere in Hobart, Tasmania.

In addition to the moored sensors, surface water samples were collected at 1–2 month intervals from the Australian National Reference Stations^46^ (NRS) for KAI and MAI sites and from approximately 6 monthly CTD stations at SOTS. These samples were analysed for DIC by coulometry, and for A_T_ by potentiometric titration^47^. Certified reference materials and duplicate samples confirmed that the accuracy and precision of both measurements were ±2 μmol/kg. A stepwise linear regression of measured A_T_ values against S and T was used to establish a best fit linear equation to estimate A_T_. Including T did not improve the fit and two equations with salinity resulted: A_T (KAI)_ = 566 + 50 ·S for KAI, with residuals of ±3 μmol kg^−1^ and A_T (MAI_SOTS)_ = 787 + 44 ·S for both MAI and SOTS, with residuals of ±1.9 μmol kg^−1^. MAI and SOTS sites have a similar salinity range (34.5–35.7 and 34.1–35.1, respectively), while KAI salinities are higher (35.4–36.5). The moored sensor S measurements were then used to estimate 3-hourly A_T_ values, and the A_T_ and $fC{O}_{2}^{sw}$ pair used with measured S and T in the CO2sys program^48–53^ to estimate DIC. The calculated DIC agreed to within ±3 μmol/kg with measured values.

In order to evaluate the seasonal variability, measured and estimated data from KAI and MAI were fit to a harmonic equation that simultaneously separates the long-term trend and the seasonal cycle:

1 $$\begin{array}{c}y\,(t)=\sum _{i=0}^{3}\,{a}_{i}{t}^{i}+\sum _{h=0}^{1}\,\sum _{j=\frac{1}{2},3}\,({b}_{hj}{t}^{h}\cdot \,\cos \,(j2\pi t)+{c}_{hj}{t}^{h}\cdot \,\sin \,(j2\pi t))\\ \,\,\,+\,\sum _{h=0,4}\,\sum _{j=1,2,4}\,({d}_{hj}{t}^{h}\cdot \,\cos \,(j2\pi t)+{e}_{hj}{t}^{h}\cdot \,\sin (j2\pi t))\end{array}$$

where $y(t)$ represents the predicted time series of a variable and $t$ is the time expressed as a fraction of year based on the number of data since the start and end dates of the time series. The coefficients ${a}_{i}$, ${b}_{hj}$ and ${c}_{hj}$, ${d}_{hj}$ and ${e}_{hj}$ were linearly obtained. The first term of Eq. 1 represents the data trend^54^. The sequence of five harmonics (j) represents the annual cycle^54^ and includes harmonics with constant (h = 0) and variable amplitude^54^. (h = 1, 4). The variability in the amplitude of the harmonics was considered because it increased the fit between the time series and Eq. 1. The residuals from the fit between Eq. 1 and the time series were filtered in the time domain using two different low-pass filters and then added to the function^55^. The data gap at KAI adds some uncertainty to the estimated coefficients in Eq. 1. The coefficients of determination (R^2^) of the fits between the time series and Eq. (1) for KAI and MAI were: 0.74, 0.78 for S; 0.93, 0.95, for T and 0.86, 0.87 for $fC{O}_{2}^{sw}$(see Suplementary Fig. S1). The mean seasonal variability for each variable is obtained from the second and third terms of Eq. 1, or $y\,(t)-\sum _{i=0}^{3}\,{a}_{i}{t}^{i}$.

The scarcity of the data from SOTS does not allow us to use Eq. 1 and the mean seasonal variability of the variables was obtained by estimating monthly means without considering interannual variability. The uncertainties added by using this approximation require care to be taken when considering the role of the different processes in the seasonal variability of $fC{O}_{2}^{sw}$ at SOTS.

The seasonal variability of $fC{O}_{2}^{sw}$ in the ocean surface is mainly due to changes in thermohaline properties, air-sea fluxes and to processes associated with ocean dynamics and biology:

2 $${d}_{obs}\,fC{O}_{2}^{sw}={d}_{T}fC{O}_{2}^{sw}+{d}_{S}\,fC{O}_{2}^{sw}+{d}_{air-sea}\,fC{O}_{2}^{sw}+{d}_{dyn-bio}\,fC{O}_{2}^{sw}$$

where ${d}_{obs}\,fC{O}_{2}^{sw}$ is the monthly change in $fC{O}_{2}^{sw}$ and ${d}_{T}fC{O}_{2}^{sw}$, ${d}_{S}\,fC{O}_{2}^{sw}$ and ${d}_{air-sea}\,fC{O}_{2}^{sw}$ are the monthly changes in $fC{O}_{2}^{sw}$ due to changes in sea-surface T, S and sea-air exchange, respectively. ${d}_{T}fC{O}_{2}^{sw}$ and ${d}_{S}\,fC{O}_{2}^{sw}$ were estimated assuming solution equilibria (CO_2_ sys program). The mean uncertainty (propagated) for both terms is ±4 μatm.

We used daily averaged orthogonal wind speed data from NCEP-DOE Reanalysis 2^56^ for MAI, KAI and SOTS to estimate ${d}_{air-sea}\,fC{O}_{2}^{sw}\,(\frac{F}{MLD};\,F=k\cdot s\cdot {\rm{\Delta }}fC{O}_{2})$^51,57^. For MAI and KAI the MLD on site was estimated from NRS stations using a threshold density value (0.03 kg m^−3^)^58^. We also considered the MLD immediately off-shore of these two sites on the shelf edge using a monthly climatology derived from Argo floats^58^. For SOTS, we used the MLD on site obtained from SOTS temperature sensors^59^. Although the uncertainty in the estimate of the MLD could be lower for SOTS^34^, we considered an error of 25% in the estimate of MLD for the three sites due to the higher errors of the climatology^58^. The mean uncertainty (propagated) for ${d}_{air-sea}\,fC{O}_{2}^{sw}$ is ±3 μatm, being F the major contributor.

The last term in Eq. 2, ${d}_{dyn-bio}\,fC{O}_{2}^{sw}$, accounts for the changes in $fC{O}_{2}^{sw}$ due to ocean dynamics and biological processes and is calculated as a residual term. Its values are shown in Fig. 2b–d (grey squares) and has a mean uncertainty (propagated) of ±6 μatm.

We also approximated ${d}_{dyn-bio}\,fC{O}_{2}^{sw}$ as:

3 $${d}_{dyn-bio}\,fC{O}_{2}^{sw}\approx \,{d}_{bio}\,fC{O}_{2}^{sw}+{d}_{entr}\,fC{O}_{2}^{sw}+{d}_{adv}\,fC{O}_{2}^{sw}+{d}_{dif}\,fC{O}_{2}^{sw}$$

where ${d}_{bio}\,fC{O}_{2}^{sw}$, and ${d}_{entr}\,fC{O}_{2}^{sw}$, ${d}_{adv}\,fC{O}_{2}^{sw}$ and ${d}_{dif}fC{O}_{2}^{sw}$ the effect of entrainment, advection and diffusion processes. These variables were first estimated as changes in DIC and then transformed to changes in $fC{O}_{2}^{sw}$ using the CO2sys program.

The term ${d}_{bio}\,fC{O}_{2}^{sw}$ in Eq. 3 represents the effect of the net community production (NCP) on $fC{O}_{2}^{sw}$ and was estimated from net primary production (NPP) data obtained from MODIS and the CbPM algorithm^60^. We assume the error of the NPP from the CbPM algorithm to be of ~35%^60^. NCP was used as the amount of DIC consumed by the marine organisms and transformed into a change in $fC{O}_{2}^{sw}$ using CO2sys. We assumed that NCP constitutes ~15% of NPP^61,62^ for KAI and MAI sites and 55% for SOTS site^43,61,62^. The mean uncertainty (propagated) of the term ${d}_{bio}\,fC{O}_{2}^{sw}$ is ±6 μatm and was estimated in basis of the values of NCP at SOTS site, since SOTS presents the highest variability of NCP of all three sites.

The entrainment term in Eq. 3 is estimated as ${d}_{entr}\,fC{O}_{2}^{sw}=\frac{dMLD}{dt}\cdot (DI{C}_{tc}-DIC)\,$^63^, which accounts for the exchange of DIC between the mixed layer ($DIC$) and the underlying seasonal thermocline waters ($DI{C}_{tc}$) as the mixed layer depth changes with time ($\frac{dMLD}{dt}$). We assumed that DIC, obtained from $fC{O}_{2}^{sw}$ and A_T_, was representative of the mean value in the mixed layer. For KAI and MAI, the monthly values of DIC below the MLD ($DI{C}_{tc}$) were obtained from measurements at the sites. For SOTS, we obtained averaged winter and summer values of DIC below MLD from repeats of the nearby WOCE SR03 hydrographic section^64^ and interpolated linearly to obtain monthly values. The mean uncertainty (propagated) of ${d}_{entr}\,fC{O}_{2}^{sw}$ is ±4 μatm, being the DIC terms the major contributors.

The advection term in Eq. 3 was estimated as ${d}_{adv}\,fC{O}_{2}^{sw}=\frac{1}{MLD}\cdot v\frac{dDIC}{dx}$^63^, where the mean horizontal gradient of DIC in the direction of mean flow ($\frac{dDIC}{dx}$) was obtained using surface data climatology from GLODAPv2^65^ and the monthly surface flow, $v$, was estimated using modelled flow from the B-SOSE simulation^14^. The estimated (propagated) uncertainty of ${d}_{adv}\,fC{O}_{2}^{sw}$ is ±4 μatm in average and is mostly due to the uncertainty of DIC.

The diffusion term in Eq. 3 was estimated as ${d}_{dif}fC{O}_{2}^{sw}=\frac{1}{MLD}\cdot {K}_{z}\cdot \frac{dDIC}{dz}$^63^ with vertical diffusivity coefficients of ${K}_{z}$ = 0.5 10^−4^ m^2^ s^−1^ for SOTS, ${K}_{z}$ = 0.6 10^−4^ m^2^ s^−1^ for KAI and ${K}_{z}$ = 0.8 10^−4^ m^2^ s^−1^ for MAI^66^. The vertical gradient of DIC ($\frac{dDIC}{dz}$) was obtained from the measurements at the sites for KAI and MAI and from the repeats of the WOCE SR03 hydrographic section for SOTS. The mean uncertainty (propagated) of ${d}_{dif}fC{O}_{2}^{sw}$ is ±3 μatm, being DIC the major contributor.

The interannual variability of $fC{O}_{2}^{sw}$ was only analysed for KAI and MAI because of the discontinuity in SOTS data. The interannual trend for the period 2012–2016 was obtained from the first term of Eq. 1. The effect of temperature ${d}_{T}fC{O}_{2}^{sw}$ and salinity ${d}_{S}fC{O}_{2}^{sw}$ on the interannual change of $fC{O}_{2}^{sw}$ between 2012 and 2016 was examined using the CO2sys program by having the correspondent variable follow the observed changes while keeping all other variables constant. Monthly differences between 2016 and 2012 in $fC{O}_{2}^{sw}$, temperature and salinity were also computed.

## Supplementary information

Supplementary material

## Data Availability

The KAI and MAI data are available through Australia’s Integrated Marine Observing System data portal (https://portal.aodn.org.au). The SOTS CO_2_ data are available via the NOAA National Centers for Environmental Information data portal (https://www.nodc.noaa.gov/ocads/oceans/Moorings/SOFS.html). Data from other SOTS sensors are available through Australia’s Integrated Marine Observing System data portal (https://portal.aodn.org.au).
