# Supplementary material for: Surface ocean carbon dioxide variability in South Pacific boundary currents and Subantarctic waters
Source: Sci Rep. 2019 May 20;9:7592. doi: 10.1038/s41598-019-44109-2 (PMC6527708; doi:10.1038/s41598-019-44109-2)
Supplement: Supplementary file 1 — Supplementary material [file 41598_2019_44109_MOESM1_ESM.pdf]

SUPPLEMENTARY INFORMATION

Surface ocean carbon dioxide variability in South Pacific boundary currents and Subantarctic waters.

Paula C. Pardo[1*], Bronte Tilbrook[1,2], Erik van Ooijen[2+], Abraham Passmore[2+], Craig Neill[2+], Peter Jansen[2], Adrienne J. Sutton[3+], Thomas W. Trull[1,2+]

[1]Antarctic Climate and Ecosystem Cooperative Research Center (ACE-CRC), Hobart, Australia
[2]CSIRO Oceans and Atmosphere, Hobart, Australia
[3]National Oceanic and Atmospheric Administration (NOAA)/ Pacific Marine Environmental Laboratory (PMEL), Seattle, Washington, USA
[*]paula.condepardo@csiro.au/paula.condepardo@utas.edu.au, ORCID ID: https://orcid.org/0000-0001-6348-2332.
[+]these authors contributed equally to this work

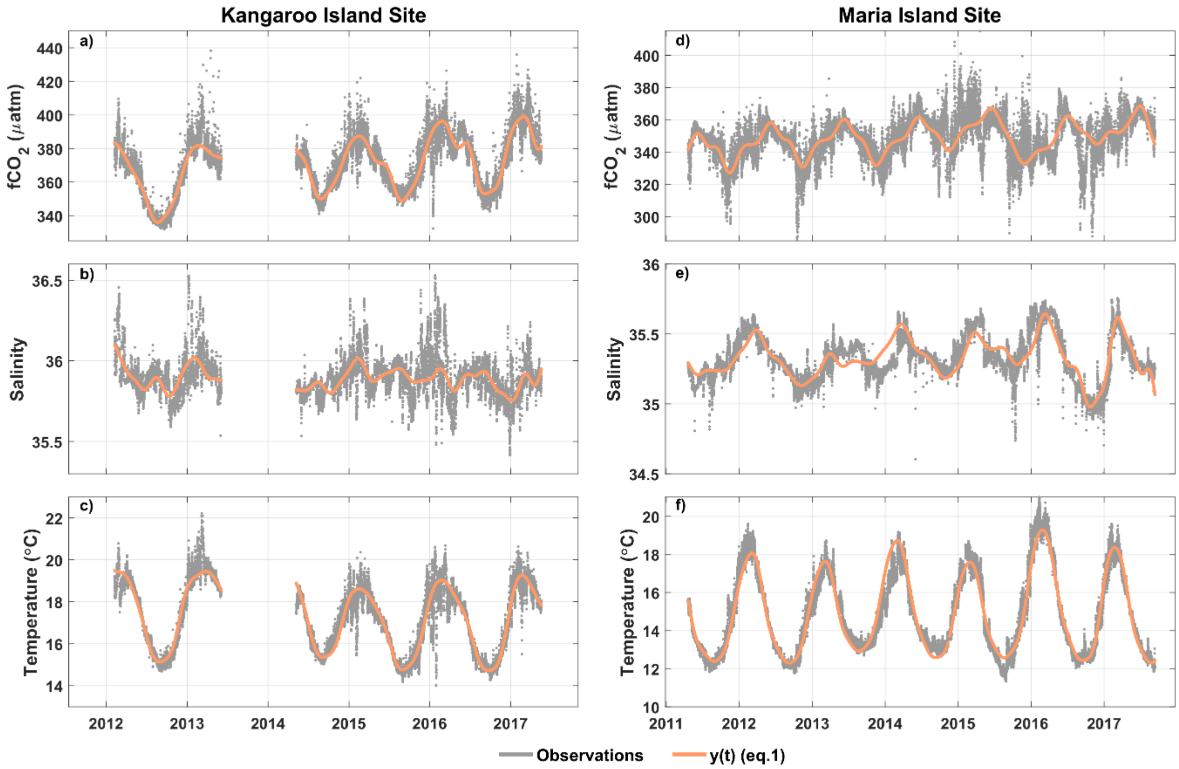

Figure S1. Results from equation 1 (y(t)) in Methods (coloured lines) compared to the observed data (grey dots) of fugacity (fCO_2), temperature and salinity at Kangaroo Island (KAI) and Maria Island (MAI) sites. The sequence of harmonics (j) in eq. 1 were selected after a Fourier analysis.
